# COVID-19 Vaccine Booster Hesitancy (VBH) of Healthcare Workers in Czechia: National Cross-Sectional Study

**DOI:** 10.3390/vaccines9121437

**Published:** 2021-12-06

**Authors:** Miloslav Klugar, Abanoub Riad, Lekshmi Mohanan, Andrea Pokorná

**Affiliations:** 1 Czech National Centre for Evidence-Based Healthcare and Knowledge Translation (Cochrane Czech Republic, Czech EBHC: JBI Centre of Excellence, Masaryk University GRADE Centre), Faculty of Medicine, Institute of Biostatistics and Analyses, Masaryk University, 625 00 Brno, Czech Republic; klugar@med.muni.cz (M.K.); lekshmi.mohanan@mail.muni.cz (L.M.); apokorna@med.muni.cz (A.P.); 2 Department of Health Sciences, Faculty of Medicine, Masaryk University, 625 00 Brno, Czech Republic; 3 Institute of Health Information and Statistics of the Czech Republic, 128 01 Prague, Czech Republic; 4 Department of Public Health, Faculty of Medicine, Masaryk University, 625 00 Brno, Czech Republic

**Keywords:** booster immunization, COVID-19 vaccines, Czechia, decision making, health personnel

## Abstract

The emerging SARS-CoV-2 variants and waning vaccine-elicited immunity are two public health challenges that occurred simultaneously and synergistically during the summer of 2021 and led to a surging demand for COVID-19 vaccine booster dose (BD) rollout. This study aimed to evaluate the COVID-19 vaccine booster hesitancy (VBH) among Czech healthcare workers to explore the potential determinants of VBH. A national cross-sectional survey-based study was carried out between 3 and 11 November 2021, using an online self-administered questionnaire (SAQ) that explored the participants’ demographic characteristics, COVID-19 infection and vaccine anamneses, willingness to receive COVID-19 vaccine BD, and the psychosocial drivers of VBH. A total of 3454 HCW properly responded to the online SAQ, of which 80.9% were females, 30.3% were medical professionals, and 50.5% were ≤47 years old. Most of the participants were already inoculated against SARS-CoV-2 (95.2%), and BTN162b2 was the most commonly administered vaccine (90.7%). As the study sample was planned to represent the target population, it revealed a high level of BD acceptance (71.3%) among Czech HCW, while 12.2% were still hesitant and 16.6% were against the currently available BD. These results are consistent with other recent results from central Europe. Medical professional, male, and older participants were more likely to accept BD rather than allied health professional, female, and younger participants. The BDs’ perceived effectiveness against severe illness, symptomatic infection, and community transmission was a significant and strong predictor for BD acceptance, while the effectiveness against the circulating variants was not that important for our target population. The BDs’ perceived safety and ethical dilemmas of vaccine justice should be addressed sufficiently while communicating with HCW and other population groups. The altruistic reasons for BD acceptance, i.e., family protection, patient protection, and community health protection, underpin the recommendation of postponing the COVID-19 vaccine mandating in favour of stressing these altruistic concerns amid public health messaging.

## 1. Introduction

Healthcare workers (HCW) have experienced disproportionately high levels of COVID-19-associated morbidity and mortality; therefore, they were prioritized for receiving COVID-19 vaccine booster doses (BDs), which are the third doses of two-dose vaccines, such as BNT162b2 and mRNA-1273, or the second doses of 1-dose vaccines such as Ad26.COV2.S, along with those aged above 65 and the immunocompromised population [1,2]. Since the epidemic flare-ups and effective vaccines’ accessibility are long-standing challenges, distrust of vaccines’ BDs has been increasingly reported, especially in the last few years, triggered by anti-vaccination campaigns [3,4,5].

Recent reports have been increasingly suggesting that the effectiveness of COVID-19 vaccines had declined in several countries within 6 months after the primer doses’ rollout [6,7,8,9]. The recorded epidemic flare-ups and breakthrough cases had been attributed to two possibilities; (a) the emerging variants (mutations) of the severe acute respiratory syndrome coronavirus 2 (SARS-CoV-2), or (b) the waning vaccine-elicited immunity that is challenged by the heightened immune evasion by the circulating variants, e.g., the Delta variant, which were not known at the time of the vaccine development and can partially bypass the protective mechanisms established after vaccination and cause illness, leaving the vaccinated cohorts vulnerable [7,8,9,10,11,12]. As both phenomena occur simultaneously and synergistically, the duty of epidemiologic and public health researchers to clarify this foggy scene and provide evidence-informed recommendations regarding the COVID-19 vaccines’ BD timing and priority groups became a challenging one.

It was clearly evident in population-level studies from Scotland, the United States, and Qatar that protection from COVID-19 symptomatic infection can be expected from the two-dose vaccination regimes that were effective against the Delta variant (B.1.617.2) [13,14,15]. Likewise, protection from severe illness can also be achieved according to the population studies of Canada, Scotland, and the United Kingdom, especially against the Alpha variant (B.1.1.7) [9,13,16]. However, the same studies noted a slight drop in the effectiveness of COVID-19 vaccines to around 80%; the sustained reduction in infection risk underscores the continued importance and benefits of COVID-19 vaccination [17]. The good news is that further population studies showed a significant decrease in relative risk of symptomatic infection and severe illness in the patients who received a COVID-19 vaccine BD [18,19]. Therefore, the rollout of BDs -including COVID-19 vaccine BDs- has always been required to battle some of the deadliest diseases, e.g., Hepatitis B and pertussis, which are seen most commonly among HCWs due to their frequent use exposure [20,21].

Vaccine hesitancy (VH) is defined by the World Health Organization (WHO) Strategic Advisory Group of Experts on Immunization (SAGE) as “delay in acceptance or refusal of vaccination despite the availability of vaccination services” [22]. COVID-19 vaccine BD hesitancy (VBH) can be predicted in populations with suboptimal vaccine uptake, such as in Poland, where only 51% of the population received a first dose until the middle of September 2021, despite the extensive scientific communication efforts and the worsening epidemic situation in the country in 2021 [23].

Vaccine selectivity (VS) is another emerging public health challenge which can be defined as “the discriminatory attitudes towards certain types of vaccines based on their target contagion or manufacturing technology that yields heterogeneous acceptance levels of recommended vaccines,” and can be simply understood as the individual’s or public’s preference of particular vaccine/s based on their mode of action or brand rather than general preference of immunization [24]. This phenomenon has been commonly seen with childhood vaccines, as parents can be selective with certain vaccines that they believe effective and/or safe for their children, and decline other vaccines [25]. A similar situation had been reportedly experienced with COVID-19 vaccines due to the diversity of vaccine options in many countries, which created a pseudo-competitive ecology for comparing and choosing among the available vaccine brands in the market [26].

An average vaccinee may accept or reject a BD due to various triggers, including; the side effects experienced after the previous (primer) doses, perceived effectiveness of the BD, the vaccinee’s susceptibility to the target infection, and safety uncertainties [23,27]. The WHO-SAGE depicted the perceived effectiveness, safety, and susceptibility and the cost-benefit ratio evaluation as critical determinants for VH, especially for the childhood vaccines which are conventionally recommended worldwide [28,29,30,31].

The safety of COVID-19 vaccines as novel pharmacologic products is a chief priority for health systems that aim to keep tracking them during the post-marketing (phase IV) period through their passive surveillance systems, e.g., VAERS, CAEFISS, and MHRA-Yellow Cards [32,33,34]. In addition, active surveillance studies and hybrid systems had been strongly advocated, especially with the accelerated rollout of COVID-19 vaccines [35,36,37,38]. However, there is still a lack of peer-reviewed evidence on COVID-19 vaccines’ BD side effects; the preliminary findings from passive surveillance systems and the population study of Israel suggest that the BD side effects are not different from the primer doses side effects [18,39,40]. The primer doses were found to induce mild side effects in the vast majority of vaccinees, which lasted for 1–3 days on average in most of COVID-19 vaccines types, i.e., mRNA-based, viral vector-based, and inactivated virus vaccines [24,41,42,43,44,45,46,47,48].

The risk of waning immunity and development of suboptimal immune response after vaccination in older and immunocompromised people, e.g., oncologic patients, organ transplant recipients, and HIV patients, even after two-dose regimens, led to the consensus recommendation of prioritizing these high-risk groups for COVID-19 vaccine BD in various countries, e.g., the United States and France [12,49,50,51,52]. HCWs as a defined high-risk group for COVID-19 infection were also prioritized in all countries that started the rollout of COVID-19 vaccine BDs in late summer 2021, including the Czech Republic [2].

The holistic aim of this study was to evaluate the COVID-19 VBH among HCWs in Czechia. The primary objective was to assess the levels of COVID-19 vaccine BD acceptance and hesitancy among HCWs, and the secondary objective was to explore the potential demographic, anamnestic, and psychosocial determinants of the COVID-19 VBH among the target population.

## 2. Materials and Methods

### 2.1. Design

An analytical survey-based cross-sectional study was carried out between 3–11 November 2021 to explore the attitudes of Czech healthcare workers towards receiving the BD (third) doses of COVID-19 vaccines. The study utilized a self-administered questionnaire (SAQ) which was coded and disseminated online through KoBoToolbox (Harvard Humanitarian Initiative, Cambridge, MA, USA, 2021) for data collection from the target participants [53]. The Strengthening the Reporting of Observational Studies in Epidemiology (STROBE) guidelines for cross-sectional studies governed the design, conduction, and reporting of this study [54].

### 2.2. Participants

As a nationwide study, the target population (healthcare workers) was approached through various channels, aiming to achieve a nationally representative sample. A non-random technique (snowballing strategy) was used to recruit the participants from all the fourteen regions of the Czech Republic. On 1 November 2021, invitations were sent to the chairs of the professional medical societies, which are members of the Czech Medical Association of J. E. Purkyně (CzMA; Prague, Czech Republic), the member institutions coordinators of the Czech Clinical Research Infrastructure Network (CZECRIN; Brno, Czech Republic), and the inpatient healthcare facilities managers within the network of the Central Adverse Events Reporting System of the Institute of Health Information and Statistics of the Czech Republic (IHIS-CR; Prague, Czech Republic) in order to facilitate participation in the study by circulating the survey uniform resource locator (URL) through their respective networks [55,56,57]. Another channel was used in collaboration with the Ministry of Health to also include healthcare providers for inpatient long-term care; the professional association of non-medical healthcare workers, i.e., Czech Nursing Association (ČAS) [58], Union of Physical Therapists of the Czech Republic (UNIFY-ČR) [59], Chamber of Midwives of the Czech Republic (ČKPA) [60], Association of College Nurses (SVVS) [61], Association of Higher Education Educators in the Non-Medical Health Professions (AVVNZP) [62], and Association of Social Service Providers of the Czech Republic (APSS-ČR) [63]. The study was further promoted through the official websites of professional medical and allied healthcare societies, in addition to the website of the Czech Ministry of Health (MoH) [64].

Epi-Info ^TM^ version 7.2.4 (CDC. Atlanta, GA, USA, 2020) was used to calculate the optimal sample size required for this study [65]. The population survey module was run following the assumptions of 2% margin of error, 95% confidence level (CI), 50% outcome probability, and a target population size of 257,118 based on the latest report of IHIS-CR [66,67]. The required sample size was 2379 participants (Supplementary Figure S1). 

Participation in this study was entirely voluntary, thus implying that the participants were not forced or rewarded to participate. Additionally, participation in this study was anonymous, to give the participants room to freely express their views and attitudes towards vaccination, aiming to eliminate Hawthorne bias.

**Figure 1:**
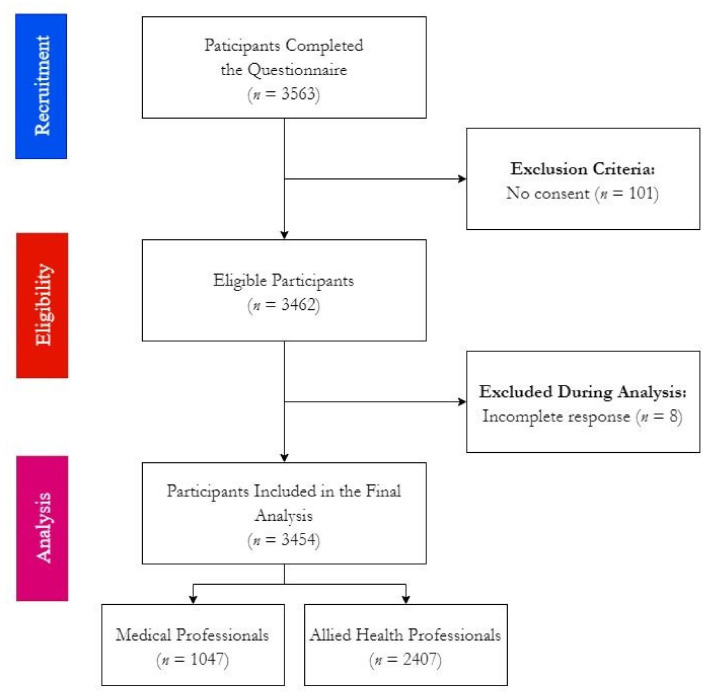
Workflow of COVID-19 Vaccine BD Survey among Czech Healthcare Workers, November 2021 (*n* = 3454).

Until 11 November 2021, 3563 responses were received, of which 101 were excluded because the participants did not consent to participate in the study after reading the informed consent, constituting 2.8% as a non-repose rate. Later, eight responses were removed due to incomplete data, leading to a final sample of 3454 participants being included in the final analyses (Figure 1).

### 2.3. Instrument

The draft SAQ was adapted from previous studies of COVID-19 vaccine hesitancy and consisted of 19 close-ended items stratified into five sections [68,69,70,71,72,73,74,75]. The first section, demographic characteristics, included gender, age, profession, and region. The second section, COVID-19-related anamnesis, included the history of COVID-19 infection, onset, clinical severity, and manifestations of the infection. The third section, COVID-19 vaccine-related anamnesis, included the history of COVID-19 vaccination, vaccine type, and the number of doses. The fourth section included a five-point Likert scale item assessing the willingness to receive BD of COVID-19 vaccines, ranging from “Totally Disagree = 1” to “Totally Agree = 1”.

The fifth section had a set of potential psychosocial drivers of COVID-19 vaccine BD acceptance, including (a) perceived effectiveness: preventing severe illness, symptomatic infection, and community transmission and controlling variants (mutations), (b) perceived safety: equal safety profile compared to primer doses, and seriousness of side effects, (c) perceived susceptibility and risk-benefit ratio, (d) moral dilemma of vaccine justice, and (e) vaccine primer dose satisfaction and vaccine selectivity.

The content validity of the draft SAQ was assessed by a panel of experts in public health medicine, health policy, and healthcare management who provided their feedback on relevance appropriateness and clarity of the proposed items. The experts’ comments guided the development of the pre-test version of the SAQ which was sent to target volunteers who filled it twice with a 14-day interval. The test-re-test reliability of the SAQ yielded a mean Cohen’s kappa coefficient of 0.80 ± 0.19 (IQR: 0.60–1.00), indicating a substantial level of reliability according to McHugh’s criteria [76] (Supplementary Table S1). 

### 2.4. Ethics

The declaration of Helsinki for research involving human subjects guided the design and execution of this study [77]. The study protocol was thoroughly reviewed by the Ethics Committee of the Faculty of Medicine at Masaryk University on 19 October 2021 under the identifier Ref. 63/2021.

All participants had to provide their informed consent as a prerequisite to taking part in the study. The participants were offered to leave the study at any time without justifying their decision; also, no data was saved before the participants finalized the questionnaire and confirmed submitting their answers. No identifying personal data were collected from the participants, in order to keep the study as much anonymous as possible and aiming for the elimination of the Hawthorne bias. The European Union (EU) General Data Protection Regulation (GDPR) was followed during data collection and processing [78].

### 2.5. Statistics

Statistical analyses were performed using the Statistical Package for the Social Sciences (SPSS) version 28.0 (SPSS Inc. Chicago, IL, USA, 2020) [79]. Initially, the normal distribution of numerical variables, e.g., age, was tested using the Shapiro-Wilk test. Descriptive statistics were performed to present all the study variables; nominal variables, e.g., gender, profession and discrete events, and ordinal variables, e.g., psychosocial drivers, had been described using frequencies (*n*) and proportions (*%*). The numerical variables, e.g., age, had been described using central tendency and dispersion properties.

Consequently, inferential statistics were performed to test for the associations between independent demographic variables (profession, age group, and gender), anamnestic variables (COVID-19 infection and vaccine primer doses), psychosocial drivers, and the dependent variable of BD-related attitudes using the Chi-squared test (*χ*^2^) and Fisher’s exact test for tests with <5 predicted frequency. Eventually, univariate logistic regression was performed to evaluate the odds ratio of vaccine hesitancy vs. acceptance for each significant demographic variable. Multivariate regression analysis of the proposed psychosocial drivers was adjusted for gender, pregnancy, age, profession, COVID-19 infection and vaccination, and seeking medical care. All inferential tests were run with the following assumptions; a confidence level (*CI*) of 95% and significance level (*Sig.*) of ≤0.05.

## 3. Results

### 3.1. Demographic Characteristics

Out of 3454 participants, females were the majority (80.9%), followed by males (18.6%) and LGBTQ+ (0.4%). There were 25 pregnant women among the participating females, of which 36% were in the first trimester, 40% the second trimester, and 24% the third trimester. The mean age of the participants was 46.97 ± 11.78 (IQR: 39–55) years old, and the median was 47 years old Table 1.

According to Czech law, Act no. 95/2004 Coll. and Act no. 96/2004 Coll., medical professions (MP) include general medicine, dentistry, and pharmacy, while allied health professions (AHP) include all the non-medical professions which are related to the provision of health care, e.g., nursing, midwifery, and physiotherapy [80,81,82]. Physicians were the most common MP (28.4%), followed by pharmacists (1.5%) and dentists (0.4%). General nurses were the most common AHP (42%), followed by paediatric nurses (4.3%), medical laboratory technicians (4.2%), and pharmaceutical assistants (3.0%). In total, MP represented 30.3% of the participating sample, while AHP represented 69.7% (Supplementary Table S1).

**Figure 2:**
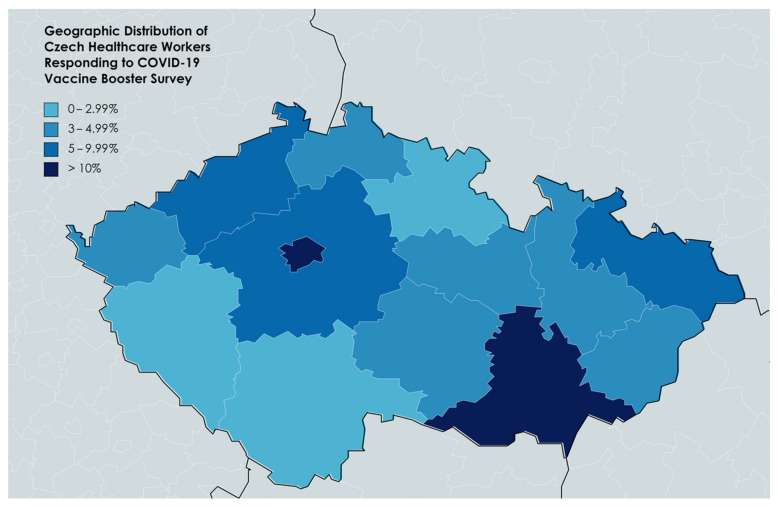
Geographic Distribution of Czech Healthcare Workers Responding to COVID-19 Vaccine BD Survey, November 2021 (*n* = 3454).

The participating sample represented all the fourteen administrative regions of the Czech Republic, Act no. 129/2000 Coll. [83]. The most contributing region was the capital city Prague (29.2%), followed by the South Moravian (20.2%), the Central Bohemian (9.8%), the Moravian-Silesian (6.0%), and the Ústecký regions (5.4%), shown in Figure 2.

### 3.2. COVID-19-Related Anamnesis

In total, 32% of the participants reported being previously infected with COVID-19, with a significant difference between MP (28.4%) vs. AHP (33.6%) and >47 years-old (29.5%) vs. ≤47 years-old (34.5%). Most infections (87.8%) occurred before receiving the first COVID-19 vaccine dose, 6.2% between the first and the second doses, and 6.0% after the second dose. According to the Australian guidelines for the clinical care of people with COVID-19, mild cases were the majority (59.5%), followed by moderate (31.4%) and asymptomatic (7.1%) [84]. There was no statistically significant difference in COVID-19 onset or severity across the profession, gender, or age group.

The most commonly reported clinical manifestation of COVID-19 infection was fatigue (77.1%), followed by myalgia (66.2%), headache (63.3%), anosmia (59.9%), fever (56.4%), cough (49.9%), and dysgeusia (46.2%). Dyspnea was significantly more common among females (30.6%) and AHP (32.8%) compared to males (21.0%) and MP (18.5%). Neurological disorders, i.e., anosmia and dysgeusia, were significantly more common among AHP, females, and the ≤47 year-old group than MP, males, and the >47 year-old group, respectively. Headache, pharyngitis, congestion, and nausea were significantly more common among females than males. Headache, nausea, and vomiting were significantly more common among AHP than MP (Table 2).

### 3.3. COVID-19 Vaccine-Related Anamnesis

The vast majority (95.2%) of the participating sample had received primer doses of COVID-19 vaccines. There were slight differences in vaccine uptake favouring MP, males, and >47 years-old compared to their counterparts. The most reportedly administered vaccine was BTN162b2 (90.7%), followed by mRNA-1273 (5.3%), AZD1222 (2.7%), and Ad26.COV2.S (1.3%). MP (95.1%) and males (92.8%) had significantly more BTN162b2 compared to AHP (88.7%) and females (92.8%). There were no gender or age-based differences in terms of the administered vaccine type.

Less than half of the sample received a third dose by the time of responding to this survey (48.5%), and the rest received either two doses (49.7%) or one dose (1.8%). MP received significantly (*Sig.* < 0.001) more three doses (60.2%) than AHP (43.3%). Likewise, males received significantly (*Sig.* < 0.001) more three doses (60.6%) than females (45.8%). Only 2.2% of the total participants reported seeking medical care following the COVID-19 vaccine primer doses; three reported anaphylaxis, 26 lymphadenopathy, 67 myalgia, 67 arthralgia, 58 fatigue, 70 fever, 17 vomiting, and 65 headache (Table 3).

### 3.4. COVID-19 Vaccine BD-Related Attitudes

**Figure 3:**
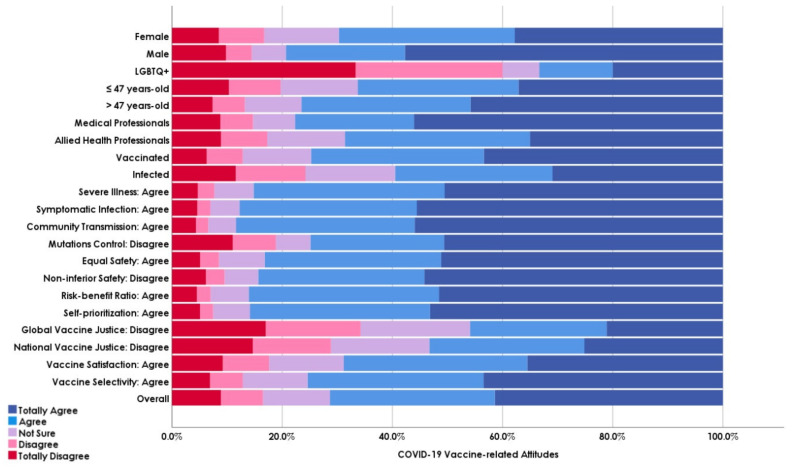
COVID-19 Vaccine BD-related Attitudes of Czech Healthcare Workers Responding to COVID-19 Vaccine BD Survey, November 2021 (*n* = 3454).

When asked about their attitudes towards receiving COVID-19 vaccine BDs, 71.3% indicated their acceptance (“totally agree” and “agree”), 12.2% were hesitant (“not sure”), and 16.5% disclosed their rejection to receive the BD (“totally disagree” and “disagree”). MP (77.7%), male (79.3%), and >47 year-old participants (76.5%) had significantly (*Sig.* < 0.001; <0.001; and <0.001) higher levels of BD acceptance than AHP (68.6%), female (69.7%), and ≤47 year-old participants (66.3%), (Figure 3).

The BD-accepting group was asked about their motivators for receiving the BD; the most common promoter was to protect their families (83.0%), followed by protecting their own health (82.7%), protecting their patients (70.4%), community health protection (66.4%), and having lesser restrictions on the social activities, e.g., travel (49.8%). Employer’s endorsement was reported as the motivator for only 3.4% of the participants.

MP had significantly (*Sig.* < 0.001; and <0.001) higher levels of interest in patients’ protection and community health protection (74.8% and 76.0%, respectively) compared to AHP (68.2% and 61.7%, respectively). Similarly, male participants had a significantly (*Sig.* < 0.001) higher level of interest in community health protection (73.7%) than their female counterparts (64.4%), (Table 4).

### 3.5. Psychosocial Drivers of BD-Related Attitudes

The perceived effectiveness of BDs was assessed through four items; (i) severe illness, (ii) symptomatic infection, (iii) community transmission, and (iv) mutation control. A total of 80.3% of the participants agreed that the current BD could protect them from severe illness, while only 57.8% agreed that BDs could prevent symptomatic infection. A total of 60.8% and 65.1% of the participants agreed that BDs could prevent community transmission and tackle the new circulating variants, respectively.

The perceived safety was assessed using two items; (i) equal safety profile and (ii) non-inferior safety (severe side effects). In total, 76.5% agreed that the current BDs are as safe as the primer doses, while only 12.5% believe that the BD may impose more severe side effects compared to the primer ones.

The perceived susceptibility was assessed using one item exploring the participants’ views of self-prioritization for BDs; 67.2% of the participants agreed to be prioritized to receive the currently available BDs, and 78.1% believed that the benefits of BDs outweigh their risks.

The moral dilemma of vaccine justice was evaluated on two levels; (i) globally and (ii) nationally. Less than one-third (30.5%) of the participants disagreed with receiving the BD after learning that administering third doses in developed economies may deprive masses in the third world from getting even the first dose. More than one-third (37.6%) of the participants disagreed with receiving the BD after learning that this may affect the accessibility of some population groups to the vaccine (Table 5).

Dissatisfaction with the primer doses type was reported by only 10.7% of the participants. More than one-fifth (21.4%) of the participants were vaccine-selective as they agreed that the government should purchase a certain vaccine type/brand for the BD, of which BTN162b2 was the most commonly recommended type (69.3%), followed by mRNA-1273 (21.9%), Ad26.COV2.S (6.5%), and AZD1222 (2.4%).

MP had significantly (*Sig*. < 0.001, <0.001 and <0.001) higher agreement with the BD capacity against severe illness, symptomatic infection, and community transmission (85.7%, 63.7%, and 66.9%) compared to AHP (77.9%, 55.3%, and 58.2%), respectively. There was no significant (*Sig.* = 0.808) difference between MP (64.8%) and AHP (65.2%) in terms of agreement with the BD capacity against mutations. MP had significantly (*Sig*. < 0.001 and <0.001) higher agreement with the equal safety of BDs and higher disagreement with the increased severity of BD side effects (84.5% and 65.8%) compared to AHP (73.0% and 51.5%), respectively.

MP had significantly (*Sig*. < 0.001, <0.001, and <0.001) higher agreement with the BD capacity against severe illness, symptomatic infection, and community transmission (85.7%, 63.7%, and 66.9%) compared to AHP (77.9%, 55.3%, and 58.2%), respectively. There was no significant (*Sig.* = 0.808) difference between MP (64.8%) and AHP (65.2%) in terms of agreement with the BD capacity against mutations. MP had significantly (*Sig*. < 0.001 and <0.001) higher agreement with the equal safety of BDs and higher disagreement with the increased severity of BD side effects (84.5% and 65.8%) compared to AHP (73.0% and 51.5%), respectively.

Moreover, MP had significantly (*Sig*. < 0.001 and <0.001) higher levels of favourable BD risk-benefit ratio and perceived susceptibility (84.1% and 72.6%) than AHP (75.4% and 64.8%), respectively. Interestingly, MP had significantly (*Sig*. < 0.001 and <0.001) lower levels of disagreement to receive BDs due to vaccine justice dilemmas globally and nationally (23.3% and 31.5%) compared to AHP (33.6% and 40.2%), respectively. There was no significant difference in terms of primer dose satisfaction or vaccine selectivity between MP and AHP.

Males had significantly (*Sig*. < 0.001, <0.001, and =0.014) higher agreement with the BD capacity against severe illness, symptomatic infection, and community transmission (86.8%, 65.8%, and 65.2%) compared to females (78.9%, 56.3%, and 59.9%), respectively. There was no significant (*Sig.* = 0.077) difference between males (62.1%) and females (65.7%) in terms of agreement with the BD capacity against mutations. Males had significantly (*Sig*. < 0.001 and <0.001) higher agreement with the equal safety of BD and higher disagreement with the increased severity of BD side effects (85.8% and 70.5%) compared to females (74.6% and 52.5%), respectively.

Additionally, males had significantly (*Sig*. < 0.001 and <0.001) higher levels of favourable BD risk-benefit ratio and perceived susceptibility (85.4% and 72.8%) than females (76.6% and 66.0%), respectively. Likewise, males had significantly (*Sig*. < 0.001 and <0.001) lower levels of disagreement to receive BD due to vaccine justice dilemmas globally and nationally (23.2% and 29.7%) compared to females (32.0% and 39.3%), respectively. All the differences between the ≤47 year-old group and the >47 year-old group were not statistically significant. Table 6.

### 3.6. Determinants of BD-Related Attitudes

All the demographic variables had impact on BD acceptance; male, non-pregnant women, MP, and >47 year-old participants had significantly (*Sig*. < 0.001, <0.001, <0.001, and <0.001) higher levels of BD acceptance (79.3%, 70.0%, 76.5%, and 77.7%) compared to female, pregnant women, AHP, and ≤47 year-old participants (69.7%, 36.0%, 66.3%, and 68.6%), respectively.

While the previously infected participants (59.5%) had a significantly (*Sig*. < 0.001) lower level of BD acceptance compared to their counterparts (76.9%), there was no significant difference due to onset, clinical severity or most of the clinical manifestations.

Regarding the vaccine-related anamnesis, the previously vaccinated participants (74.7%) had a significantly (*Sig*. < 0.001) higher level of BD acceptance compared to the non-vaccinated participants (4.2%). BTN162b2 was the vaccine type associated with highest level of BD acceptance (76.6%), while Ad26.COV2.S had the lowest level of BD acceptance (27.3%). The participants who sought medical care following their primer dose (38.1%) had a significantly (*Sig*. < 0.001) lower level of BD acceptance than their counterparts (76.1%).

The agreement with the BD capacity against severe illness, symptomatic infection, and community transmission was significantly associated (*Sig*. < 0.001, <0.001, and <0.001) with higher levels of BD acceptance (85.2%, 87.7%, and 88.4%) compared to the disagreement with these constructs (11.0%, 39.2%, and 29.6%), respectively. Contrarily, the agreement with the BD capacity against mutations (69.8%) was not significantly different from the disagreement (74.9%). Moreover, the agreement with equal safety and the disagreement with severer side effects were significantly associated (*Sig*. < 0.001 and <0.001) with BD acceptance (83.2% and 84.3%) compared to the disagreement with equal safety and the agreement with severer side effects (15.4% and 48.3%), respectively.

Likewise, the favourable risk-benefit ratio and agreement with perceived susceptibility were significantly associated (*Sig*. < 0.001 and <0.001) with higher BD acceptance (86.1% and 85.9%) compared to the unfavourable risk-benefit ratio and disagreement with the perceived susceptibility (16.0% and 33.3%), respectively. The participants who were affected by the ethical dilemmas of vaccine justice globally and nationally were significantly associated (*Sig*. < 0.001 and <0.001) with decreased levels of BD acceptance (45.9% and 53.3%) compared to the participants who were not affected by the dilemmas (87.7% and 87.6%).

The primer dose satisfaction did not impact the BD acceptance, while the vaccine selectively led to a non-significant increase in BD acceptance. The BD acceptance was the highest in BTN162b2 as the preferred vaccine type (78.1%), and the lowest in the case of Ad26.COV2.S (35.1%) (Table 7).

### 3.7. Analysis of COVID-19 Vaccine BD Hesitancy vs. Acceptance

Univariate logistic regression was performed to estimate the odds ratio (OR) of BD hesitancy and BD acceptance across the significant demographic and anamnestic predictors. Female participants were 2.36 (CI 95%: 1.69–3.31) times more likely to be BD-hesitant than males, and pregnant women were also 1.22 (CI 95%: 0.42–3.57) more likely to be hesitant compared to non-pregnant women. The young age group, AHP, the previously infected participants, the previously non-vaccinated participants, and the participants who sought medical care were 1.42 (CI 95%: 1.15–1.74), 1.99 (CI 95%: 1.54–2.57), 1.71 (CI 95%: 1.39–2.10), 1.83 (CI 95%: 1.01–3.32), and 1.75 (CI 95%: 1.08–2.82) times more likely to be hesitant compared to their counterparts.

Contrarily, male participants were 1.67 (CI 95%: 1.36–2.05) times more likely to be BD-accepting than females, and non-pregnant women were also 4.15 (CI 95%: 1.83–9.43) more likely to accept BD compared to pregnant women. The old age group, MP, the previously non-infected participants, the previously vaccinated participants, and the participants who did not seek medical care were 1.66 (CI 95%: 1.43–1.92), 1.59 (CI 95%: 1.34–1.88), 2.27 (CI 95%: 1.95–2.65), 67.66 (CI 95%: 31.62–144.81), and 5.17 (CI 95%: 3.51–7.63) times more likely to accept BDs than their counterparts (Table 8).

Multivariate logistic regression was performed to estimate the adjusted odds ratio (AOR) of BD acceptance across the various psychosocial predictors while controlling for the significant demographic and anamnestic drivers. The agreement with controlling of severe illness, symptomatic infection, and community transmission was associated with an AOR of 25.55 (CI 95%: 19.45–33.57), 5.81 (CI 95%: 4.78–7.07), and 7.90 (CI 95%: 6.47–9.65) times more likely to be BD-accepting compared to their counterparts, respectively.

The disagreement with controlling mutations and severer side effects was associated with AOR of 1.31 (CI 95%: 1.01–1.69) and 3.97 (CI 95%: 3.28–4.81) times more likely to accept BDs, respectively. The favourable risk-benefit ratio and the perceived susceptibility increased the AOR of BD acceptance with 19.42 (CI 95%: 15.20–24.80) and 7.10 (CI 95%: 5.83–8.63) times, respectively. Being influenced by the ethical dilemma of vaccine justice both globally 0.23 (CI 95%: 0.19–0.28) and nationally 0.32 (CI 95%: 0.27–0.39) was associated with a decreased AOR of BD acceptance. Vaccine satisfaction and selectivity had no significant impact on BD acceptance (Table 9).

## 4. Discussion

This study revealed that a high proportion (71.3%) of the Czech HCW favour the COVID-19 vaccine BD, while 12.2% are still hesitant and 16.6% are against the currently available BD. These results are consistent with what had been recently reported by Rzymski et al., 2021, who found that 71% of the Polish adult population declared their willingness to receive a COVID-19 vaccine BD, while the rest were not in favour of BDs [23]. In Japan, Sugawara et al., 2021 revealed that 89.1% of Japanese medical students were willing to receive the hypothetical BDs of COVID-19 vaccines [85].

The target population of this study was Czech HCW; therefore, the harvested sample was intended to be as representative as possible for the target population. According to a recent report of the Czech Statistical Office (CZSO), around 21.9% of the HCW in the Czech Republic were males, while the vast majority (78.1%) were females in 2019 [86]. Our sample reflected this gender distribution, as 18.6% were males and 80.9% were females. The mean age of Czech HCW according to the CZSO report of 2017 was 46.1 years old, thus corresponding with the mean age of our harvested sample (46.97 ± 11.78) [87]. Regarding the health professions’ categories, the latest report of IHIS-CR revealed that about 20.9% were MP and 79.1% were AHP; therefore, MP was over-represented in our sample (30.3%) compared to AHP (69.7%) [67].

Less than one-third (32%) of our participants had reported being infected by SARS-CoV-2 in the previous time. Until 10 November 2021, 1.8 million accumulated cases of COVID-19 were reported in the Czech Republic, representing 17.2% of the general population, thus making the proportion of infected participants higher than the average population [88]. According to Jarkovsky et al., 2021, around 6.2% of the confirmed COVID-19 patients during the first wave in the Czech Republic were suffering from a severe clinical form of the disease [89]. The proportion of the severe and critical cases in our sample was 2% lower than the reported proportion of accumulated hospitalized cases in the Czech Republic (≈ 7.7%) [90,91]. The vast majority of COVID-19 infection cases occurred before receiving the first dose (87.8%), and the rest occurred after the first dose (6.2%) and after the second dose (6%). Frontline HCW are amongst the high risk groups for COVID-19 infection; therefore, they were recommended to follow strict infection control measures for which they exhibited high levels of compliance in multiple locations where the transmission levels were lowered significantly [92,93,94]. Nevertheless, COVID-19 infections among HCW had been frequently reported both before and after the vaccine rollout with a clear and sustained decline in cases number after mass vaccination [95]. This finding confirms the substantial pooled effectiveness of the approved vaccines in the Czech Republic and the European Union, and it is in agreement with the previous findings of rapid decline in both asymptomatic and symptomatic COVID-19 infections among HCW following vaccine rollout in California [95,96].

Most participants (95.2%) reported being vaccinated against SARS-CoV-2, while 167 declared that they were not vaccinated at all. Gilboa et al., 2021 found that up to 97.1% of the surveyed HCW in Israel were willing to be vaccinated, while the most common reason for noncompliance with COVID-19 vaccination was pregnancy [97]. However, devastating inequalities between MP and AHP were reported at the beginning of the COVID-19 vaccine rollout; for instance, in the US, 75.1% of MP were vaccinated and only 45.6% of the AHP by March 2021; the difference was minor between our participating MP (96.7%) and AHP (94.5%) [98]. Moreover, the higher vaccine uptake rate among male and older participants may confirm what had been previously reported by the COVID-19 vaccine hesitancy studies that attempted to predict the drivers of COVID-19 VH and found that female gender and women of a young age can be determinants of lower vaccine acceptance [68,99,100]. The most commonly administered vaccine among our sample was BTN162b2 (90.7%), which was higher than its proportion among the general Czech population (82.7%) as of 15 November 2021 [101]. The second most common vaccine was mRNA-1273 (5.3%), followed by AZD1222 (2.7%) and Ad26.COV2.S (1.3%), and this exact order was found among the general population: 7.8%, 6.9%, and 2.6%, respectively [101].

Our participants’ most cited reason for accepting BD was family protection (83%), followed by self-protection (82.7%). Rutten et al., 2021 laid down a list of evidence-based strategies for addressing COVID-19 VH by clinical organizations, and one of those strategies was to focus on vaccines as an essential tool to protect one’s own and family health [102]. In a large survey-based study of hospital employees in the United States, the most common reason for accepting COVID-19 vaccination was family protection (86.7%), followed by self-protection (82.9%) [103]. Another recent study in Austria found that self-protection (60.3%) and family protection (55.3%) were the most common reasons for compliance with the COVID-19 guidelines; the investigators concluded that community health protection was also a significant altruistic reason for compliance [104]. The third most cited reason for accepting BD was patient protection (70.4%) which was even higher than community health protection (66.4%). One of the solid arguments for mandating COVID-19 vaccination of HCW is patient protection, thus aiming to fulfil the “do no harm” rule through continuing to provide care for all patients, including COVID-19 patients [105]. On comparing the high level of altruistic reasons for BD acceptance, e.g., family protection (82.7%), patient protection (70.4%), and community health protection (66.4%), to the extremely low level of the non-altruistic reasons, e.g., employer endorsement (3.4%), it becomes more evident that the recommendations of Gur-Arie et al., 2021 of implementing policies strengthening the HCW trust in vaccines and health systems should be the first step before proceeding to consider vaccination mandates [105].

The perceived effectiveness of COVID-19 vaccine BD was a significant and robust predictor of BD acceptance among our participants. The capacity to prevent severe illness, symptomatic infection, and community transmission was associated with an increased AOR of BD acceptance: 25.55, 5.81, and 7.90 times, respectively. The current body of evidence confirms the beliefs of Czech HCW who agreed that COVID-19 BD had capacity against infection-related outcomes; as the 7-day effectiveness of BDs was found to be 93% in reduction of hospitalization, 92% for severe illness, and 81% for COVID-19-related mortality when compared to the recipients of two doses five months ago [106]. Moreover, the adjusted rate ratio (ARR) of symptomatic infection was 11.3 times among the non-booster group vs. the booster group; likewise, the ARR of severe illness was 19.5 times among the non-booster group [40]. Presently, evidence is still lacking and required to verify the capacity of BD against community spread of COVID-19 infection, which might explain why the belief of BD capacity against mutations was not a potent predictor of BD acceptance in our sample.

The perceived safety of COVID-19 vaccine BDs was another significant and strong predictor of BD acceptance. While the assuring evidence on BD side effects is still preliminary and predominantly coming from non-peer-reviewed sources, our participants were inclined to believe that the BDs will have a similar safety profile of the primer doses (79.2%) and no more-severe side effects will emerge following BDs (56.9%). The male participants had higher levels of BD perceived safety compared to their female counterparts; therefore, the gender-based differences of the self-reported side effects following the primer doses could be hypothesized as one of the obstacles for females to accept BD as they were more likely bothered by the post-vaccination side effects [24,43,107,108]. It is yet unclear and requires further investigation why the MP may have higher BD perceived safety levels than the AHP. Gadoth et al., 2021 found that nurses were four times more likely to delay COVID-19 vaccination compared to physicians. Given that this finding was frequently reported with the influenza vaccine, the public health authorities were called upon to take this issue seriously because nurses are more involved in administering vaccines and are placed at the front line with patients [109,110,111,112].

The participants in the ethical conflict of accepting COVID-19 vaccine BDs while millions of people are still unvaccinated worldwide significantly lower odds of BD acceptance. On 12 November 2021, the WHO Director-General described the rollout of BDs in high-income countries (HIC) as a scandalous event [113]. “It makes no sense to give boosters to healthy adults, or to vaccinate children, when health workers, older people and other high-risk groups around the world are still waiting for their first dose,” Dr. Tedros Adhanom Ghebreyesus said [113]. One of the suggested approaches to achieve global vaccine equity is to donate excess doses to low- and middle-income countries (LMIC) by HIC through the COVAX platform, and such an approach can protect both LMIC and HIC [114]. Until 15 November 2021, the WHO-SAGE position from COVID-19 vaccine BD is not in favour of re-vaccinating HCW or the general healthy population, but it is also noteworthy that this recommendation will be re-evaluated in December 2021 [115].

The favourable risk-benefit ratio estimation was a significant and robust predictor of BD acceptance among our participants. The same finding was reported earlier among several population groups, e.g., Czech university students [70], Palestinian healthcare students [71], and American HCW [116], who were more likely to accept COVID-19 vaccine when they had favourable risk-benefit ratio assessment. This fundamental concept of an individual’s risk-benefit ratio assessment has been widely used in vaccine communication and vaccine hesitancy research, as it portrays perceived effectiveness and safety of the vaccine from one side and perceived susceptibility to the infection from another side [117]. Rey et al., 2018 used the risk-benefit balance (RBB) to evaluate VH among French parents, and it was proven as an effective method for explaining the VH of human papillomavirus, hepatitis B virus, measles, and seasonal influenza vaccines [117].

### 4.1. Strengths

To the best of the authors’ knowledge, this is the first study to evaluate the attitudes of the Czech population towards COVID-19 vaccine BD, and what is more important than estimating the general prevalence of BD acceptance is exploring the potential demographic and anamnestic determinants and the psychosocial drivers of VBH. This study used an anonymous online SAQ that aimed to give the respondents room to express their views towards vaccines without restrictions or feeling judged; therefore, we believe that the Hawthorne bias had been controlled in this design. Third, the harvested sample has an optimal size and decent representativeness, so it reflects several essential characteristics of the target population, e.g., mean age, gender distribution, and professional groups. Finally, the current study shed light on the COVID-19 infection rate, clinical severity stratification, and vaccination rate among Czech HCW.

### 4.2. Limitations

This study was limited by a number of factors, including (a) lack of information about the administered vaccine type of each dose; (b) lack of information on participants’ general medical anamnesis and BMI; (c) an insufficient number of pregnant women, LGBTQ+, and other minority groups; and (d) insufficient information on the post-vaccination experience of the participants, especially the side-effects of primer doses.

### 4.3. Implications

The results of this study imply that future research on COVID-19 VBH should explore the role of gender and age on VBH across different population groups. Our study also suggests that public health communication regarding COVID-19 BDs should benefit from the following approaches: (a) highlighting and emphasizing the evidence on BD effectiveness against severe illness, symptomatic infection, and community transmission; (b) highlighting and emphasizing the evidence on BD equal safety with the primer doses; (c) addressing the potential ethical conflicts especially those related vaccine justice; and (d) focusing on alteration of the individual’s risk-benefit ratio of BDs to become more favourable, especially among frontline HCW, e.g., nurses. Using altruistic motivators to induce vaccine uptake among HCW should be prioritized over mandating the vaccines.

## 5. Conclusions

Within the limitations of this study, a high level of BD acceptance (71.3%) was found among Czech HCW, while 12.2% were still hesitant and 16.6% were against the currently available BDs. These results are consistent with other recent results from central Europe. Medical professionals and male and older participants were more likely to accept BDs rather than allied health professionals and female and younger participants. The BDs’ perceived effectiveness against severe illness, symptomatic infection, and community transmission was found to be a significant and robust predictor for BD acceptance, while the effectiveness against the circulating variants was not that important for our target population. The BDs’ perceived safety and ethical dilemmas of vaccine justice should be addressed sufficiently while communicating with HCW and other population groups. The altruistic reasons for BD acceptance, i.e., family protection, patient protection, and community health protection, underpin the recommendation of postponing the COVID-19 vaccine mandating in favour of stressing these altruistic concerns amid public health messaging.

## Figures and Tables

**Table 1:** Demographic Characteristics of Czech Healthcare Workers Responding to COVID-19 Vaccine BD Survey, November 2021 (*n* = 3454).

Variable	Outcome	Frequency (*n*)	Percentage (%)
Gender	Female †	2796	80.9%
	Male	643	18.6%
	LGBTQ+	15	0.4%
† Pregnancy	Yes ‡	25	0.9%
	No	2771	99.1%
‡ Trimester	1st Trimester	9	36%
	2nd Trimester	10	40%
	3rd Trimester	6	24%
Age	≤47 years-old	1744	50.5%
	>47 years-old	1710	49.5%
Profession	Medical Professionals (MP)	1047	30.3%
	Allied Health Professionals (AHP)	2407	69.7%

No missing data. † Female participants. ‡ Pregnant participants.

**Table 2:** COVID-19-related Anamnesis of Czech Healthcare Workers Responding to COVID-19 Vaccine BD Survey, November 2021 (*n* = 3454).

Variable	Outcome	Medical Professionals (*n* = 1047)	Allied Health Professionals (*n* = 2407)	*Sig.*	Female (*n* = 2796)	Male (*n* = 643)	*Sig.*	≤47 Years (*n* = 1744)	>47 Years (*n* = 1710)	*Sig.*	Total (*n* = 3454)
**Infection**	Yes †	297 (28.4%)	809 (33.6%)	**0.002**	908 (32.5%)	195 (30.3%)	0.293	602 (34.5%)	504 (29.5%)	**0.001**	1106 (32.0%)
	No	750 (71.6%)	1598 (66.4%)		1888 (67.5%)	448 (69.7%)		1142 (65.5%)	1206 (70.5%)		2348 (68.0%)
† **Onset**	Before 1st Dose	257 (86.5%)	714 (88.3%)	0.437	793 (87.3%)	175 (89.7%)	0.352	527 (87.5%)	444 (88.1%)	0.779	971 (87.8%)
	Between Doses	22 (7.4%)	47 (5.8%)	0.330	57 (6.3%)	12 (6.2%)	0.948	41 (6.8%)	28 (5.6%)	0.390	69 (6.2%)
	After 2nd Dose	18 (6.1%)	48 (5.9%)	0.937	58 (6.4%)	8 (4.1%)	0.222	34 (5.6%)	32 (6.3%)	0.624	66 (6.0%)
† **Severity**	Asymptomatic	17 (5.7%)	62 (7.7%)	0.267	61 (6.7%)	18 (9.2%)	0.217	39 (6.5%)	40 (7.9%)	0.348	79 (7.1%)
	Mild	188 (63.3%)	470 (58.1%)	0.118	542 (59.7%)	114 (58.5%)	0.751	392 (65.1%)	266 (52.8%)	**<0.001**	658 (59.5%)
	Moderate	84 (28.3%)	263 (32.5%)	0.179	290 (31.9%)	56 (28.7%)	0.379	164 (27.2%)	183 (36.3%)	**0.001**	347 (31.4%)
	Severe	5 (1.7%)	13 (1.6%)	1.000 *	13 (1.4%)	5 (2.6%)	0.344 *	6 (1.0%)	12 (2.4%)	0.070	18 (1.6%)
	Critic	3 (1.0%)	1 (0.1%)	0.062 *	2 (0.2%)	2 (0.1%)	0.146 *	1 (0.2%)	3 (0.6%)	0.336 *	4 (0.4%)
† **Sign &** **Symptoms**	Fever	185 (62.3%)	439 (54.3%)	0.017	502 (55.3%)	119 (61.0%)	0.143	331 (55.0%)	293 (58.1%)	0.292	624 (56.4%)
	Cough	149 (50.2%)	403 (49.8%)	0.917	461 (50.8%)	90 (46.2%)	0.242	293 (48.7%)	259 (51.4%)	0.368	552 (49.9%)
	Dyspnea	55 (18.5%)	265 (32.8%)	**<0.001**	278 (30.6%)	41 (21.0%)	**0.007**	160 (26.6%)	160 (31.7%)	0.059	320 (28.9%)
	Fatigue	229 (77.1%)	624 (77.1%)	0.992	705 (77.6%)	145 (74.4%)	0.322	458 (76.1%)	395 (78.4%)	0.366	853 (77.1%)
	Myalgia	194 (65.3%)	538 (66.5%)	0.713	601 (66.2%)	128 (65.6%)	0.883	406 (67.4%)	326 (64.7%)	0.334	732 (66.2%)
	Headache	167 (56.2%)	533 (65.9%)	**0.003**	601 (66.2%)	97 (49.7%)	**<0.001**	394 (65.4%)	306 (60.7%)	0.104	700 (63.3%)
	Anosmia	163 (54.9%)	500 (61.8%)	**0.037**	574 (63.2%)	87 (44.6%)	**<0.001**	398 (66.1%)	265 (52.6%)	**<0.001**	663 (59.9%)
	Dysgeusia	115 (38.7%)	396 (48.9%)	**0.002**	444 (48.9%)	65 (33.3%)	**<0.001**	296 (49.2%)	215 (42.7%)	**0.031**	511 (46.2%)
	Pharyngitis	54 (18.2%)	148 (18.3%)	0.966	177 (19.5%)	25 (12.8%)	**0.029**	110 (18.3%)	92 (18.3%)	0.994	202 (18.3%)
	Congestion	104 (35.0%)	261 (32.3%)	0.388	319 (35.1%)	46 (23.6%)	**0.002**	228 (37.9%)	137 (27.2%)	**<0.001**	365 (33.0%)
	Rhinitis	100 (33.7%)	256 (31.6%)	0.523	301 (33.1%)	55 (28.2%)	0.180	208 (34.6%)	148 (29.4%)	0.066	356 (32.2%)
	Nausea	29 (9.8%)	128 (15.8%)	**0.011**	142 (15.6%)	14 (7.2%)	**0.002**	74 (12.3%)	83 (16.5%)	**0.048**	157 (14.2%)
	Vomiting	6 (2.0%)	49 (6.1%)	**0.006**	49 (5.4%)	6 (3.1%)	0.177	29 (4.8%)	26 (5.2%)	0.795	55 (5.0%)
	Diarrhea	46 (15.5%)	159 (19.7%)	0.114	173 (19.1%)	31 (15.9%)	0.303	96 (15.9%)	109 (21.6%)	**0.015**	205 (18.5%)

Chi-squared test (*χ*^2^) and Fisher’s exact test (*) had been used with a significance level (*Sig*.) ≤ 0.05. † COVID-19 Infection. The significant associations are in **bold** font.

**Table 3:** COVID-19 Vaccine-related Anamnesis of Czech Healthcare Workers Responding to COVID-19 Vaccine BD Survey, November 2021 (*n* = 3454).

Variable	Outcome	Medical Professionals (*n* = 1047)	Allied Health Professionals (*n* = 2407)	*Sig.*	Female (*n* = 2796)	Male (*n* = 643)	*Sig.*	≤47 Years (*n* = 1744)	>47 Years (*n* = 1710)	*Sig.*	Total (*n* = 3454)
**Vaccinated**	Yes †	1012 (96.7%)	2275 (94.5%)	**0.007**	2651 (94.8%)	625 (97.2%)	**0.010**	1637 (93.9%)	1650 (96.5%)	**<0.001**	3287 (95.2%)
	No	35 (3.3%)	132 (5.5%)		145 (5.2%)	18 (2.8%)		107 (6.1%)	60 (3.5%)		167 (4.8%)
† **Vaccine Type**	BTN162b2	962 (95.1%)	2018 (88.7%)	**<0.001**	2390 (90.2%)	580 (92.8%)	**0.041**	1481 (90.5%)	1499 (90.8%)	0.710	2980 (90.7%)
	mRNA-1273	19 (1.9%)	156 (6.9%)	**<0.001**	155 (5.8%)	20 (3.2%)	**0.008**	100 (6.1%)	75 (4.5%)	**0.046**	175 (5.3%)
	AZD1222	20 (2.0%)	68 (3.0%)	0.097	70 (2.6%)	17 (2.7%)	0.911	36 (2.2%)	52 (3.2%)	0.091	88 (2.7%)
	Ad26.COV2.S	11 (1.1%)	33 (1.5%)	0.402	36 (1.4%)	8 (1.3%)	0.879	20 (1.2%)	24 (1.5%)	0.561	44 (1.3%)
† **Doses Number**	One	16 (1.6%)	43 (1.9%)	0.539	50 (1.5%)	9 (1.4%)	0.451	29 (1.8%)	30 (1.8%)	0.918	59 (1.8%)
	Two	387 (38.2%)	1246 (54.7%)	**<0.001**	1388 (52.4%)	237 (37.9%)	**<0.001**	907 (55.4%)	726 (44.0%)	**<0.001**	1633 (49.7%)
	Three	609 (60.2%)	986 (43.3%)	**<0.001**	1213 (45.8%)	379 (60.6%)	**<0.001**	701 (42.8%)	894 (54.2%)	**<0.001**	1595 (48.5%)
† **Medical Care**	Yes	23 (2.3%)	90 (4.0%)	**0.014**	98 (3.7%)	13 (2.1%)	**0.044**	64 (3.9%)	49 (3.0%)	0.139	113 (3.4%)
	No	989 (97.7%)	2185 (96.0%)		2553 (96.3%)	612 (97.9%)		1573 (96.1%)	1601 (97.0%)		3174 (96.6%)

Chi-squared test (*χ*^2^) had been used with a significance level (*Sig*.) ≤ 0.05. † COVID-19 Vaccination. The significant associations are in **bold** font.

**Table 4:** COVID-19 Vaccine-related Attitudes of Czech Healthcare Workers Responding to COVID-19 Vaccine BD Survey, November 2021 (*n* = 3454).

Variable	Outcome	Medical Professionals (*n* = 1047)	Allied Health Professionals (*n* = 2407)	*Sig.*	Female (*n* = 2796)	Male (*n* = 643)	*Sig.*	≤47 Years (*n* = 1744)	>47 Years (*n* = 1710)	*Sig.*	Total (*n* = 3454)
**Attitudes**	Rejection	154 (14.7%)	416 (17.3%)	0.061	468 (16.7%)	93 (14.5%)	0.159	344 (19.7%)	226 (13.2%)	**<0.001**	570 (16.5%)
	Hesitancy	80 (7.6%)	340 (14.1%)	**<0.001**	379 (13.6%)	40 (6.2%)	**<0.001**	244 (14.0%)	176 (10.3%)	**<0.001**	420 (12.2%)
	Acceptance †	813 (77.7%)	1651 (68.6%)	**<0.001**	1949 (69.7%)	510 (79.3%)	**<0.001**	1156 (66.3%)	1308 (76.5%)	**<0.001**	2464 (71.3%)
† **Promoter**	Self-protection	773 (95.1%)	1511 (91.5%)	**0.001**	1806 (92.7%)	473 (92.7%)	0.949	1068 (92.4%)	1216 (93.0%)	0.582	2284 (82.7%)
	Patient Prot.	608 (74.8%)	1126 (68.2%)	**<0.001**	1376 (70.6%)	354 (69.4%)	0.601	817 (70.7%)	917 (70.1%)	0.758	1734 (70.4%)
	Family Prot.	690 (84.9%)	1354 (82.0%)	0.076	1626 (83.4%)	414 (81.2%)	0.229	996 (86.2%)	1048 (80.1%)	**<0.001**	2044 (83.0%)
	Community Prot.	618 (76.0%)	1018 (61.7%)	**<0.001**	1256 (64.4%)	376 (73.7%)	**<0.001**	788 (68.2%)	848 (64.8%)	0.080	1636 (66.4%)
	Avoid Testing	238 (29.3%)	482 (29.2%)	0.967	561 (28.8%)	158 (31.0%)	0.332	344 (29.8%)	376 (28.7%)	0.582	720 (29.2%)
	Easier Mobility	419 (51.5%)	809 (49.0%)	0.236	959 (49.2%)	267 (52.4%)	0.206	603 (52.2%)	625 (47.8%)	**0.030**	1128 (49.8%)
	Employer	26 (3.2%)	58 (3.5%)	0.685	61 (3.1%)	23 (4.5%)	0.127	38 (3.3%)	46 (3.5%)	0.754	84 (3.4%)

Chi-squared test (*χ*^2^) had been used with a significance level (*Sig*.) ≤ 0.05. Prot. refers to protection. † COVID-19 Vaccine Booster Accepting-Group. The significant associations are in **bold** font.

**Table 5:** Drivers of COVID-19 Vaccine-related Attitudes among Czech Healthcare Workers Responding to COVID-19 Vaccine BD Survey, November 2021 (*n* = 3454).

Variable	Outcome	Frequency (*n*)	Percentage (%)
[Severe Illness] I think the currently available BD (third shots) can protect me from severe COVID-19 infection.	Disagreement	309	8.9%
	Not Sure	373	10.8%
	Agreement	2772	80.3%
[Symptomatic Infection] I think the currently available BD (third shots) can protect me from symptomatic COVID-19 infection.	Disagreement	663	19.2%
	Not Sure	793	23.0%
	Agreement	1998	57.8%
[Community Transmission] I think the currently available BD (third shots) can prevent community transmission of SARS-CoV-2 and its variants.	Disagreement	645	18.7%
	Not Sure	709	20.5%
	Agreement	2100	60.8%
[Mutations Control] I will not take the third shoot (BD) until I find reliable evidence confirming their ability to tackle the new circulating variants of SARS-CoV-2.	Disagreement	589	17.1%
	Not Sure	618	17.9%
	Agreement	2247	65.1%
[Equal Safety] I think the currently available BD (third shots) are as safe as the previous doses of COVID-19 vaccines.	Disagreement	241	7.0%
	Not Sure	570	16.5%
	Agreement	2643	76.5%
[Non-inferior Safety] I think that the currently available BD (third shots) are as safe as the previous doses of COVID-19 vaccines.	Disagreement	1929	55.8%
	Not Sure	1094	31.7%
	Agreement	431	12.5%
[Risk-benefit Ratio] I believe that the benefits of BD (third shots) outweigh their risks.	Disagreement	331	9.6%
	Not Sure	426	12.3%
	Agreement	2697	78.1%
[Self-prioritization] I agree to be prioritized to receive the currently available BD (third shorts).	Disagreement	625	18.1%
	Not Sure	509	14.7%
	Agreement	2320	67.2%
[Global Vaccine Justice] I agree to receive the BD (third shot) of the COVID-19 vaccine even after learning that administering third shots in developed economies may deprive masses in the third world from getting even the first dose.	Disagreement	1052	30.5%
	Not Sure	1255	36.3%
	Agreement	1147	33.2%
[National Vaccine Justice] I agree to receive the BD (third shot) of the COVID-19 vaccine even after learning that this may affect the accessibility of some population groups to the vaccine.	Disagreement	1297	37.6%
	Not Sure	1153	33.4%
	Agreement	1004	29.1%
[Vaccine Satisfaction] I think I should receive a different vaccine type/brand for the BD from the previous doses.	Disagreement	1405	40.7%
	Not Sure	1680	48.6%
	Agreement †	369	10.7%
[Vaccine Selectivity] I think the government should purchase a particular vaccine type/brand for the BD.	Disagreement	1071	31.0%
	Not Sure	1644	47.6%
	Agreement †	739	21.4%
† [Preferred Vaccine] Which vaccine type should be promoted for BD?	BTN162b2	611	69.3%
	mRNA-1273	193	21.9%
	AZD1222	21	2.4%
	Ad26.COV2.S	57	6.5%

No missing data. † Agreement with “Vaccine Satsification” or “Vaccine Selectivity” statements.

**Table 6:** Drivers of COVID-19 Vaccine-related Attitudes among Czech Healthcare Workers Responding to COVID-19 Vaccine BD Survey Stratified by Profession, Gender, and Age Group, November 2021 (*n* = 3454).

Variable	Outcome	Medical Professionals (*n* = 1047)	Allied Health Professionals (*n* = 2407)	*Sig.*	Female (*n* = 2796)	Male (*n* = 643)	*Sig.*	≤47 Years (*n* = 1744)	>47 Years (*n* = 1710)	*Sig.*
**Severe Illness**	Disagree	78 (7.4%)	231 (9.6%)	**0.042**	261 (9.3%)	43 (6.7%)	0.033	194 (11.1%)	115 (6.7%)	**<0.001**
	Not Sure	72 (6.9%)	301 (12.5%)	**<0.001**	329 (11.8%)	42 (6.5%)	**<0.001**	210 (12.0%)	163 (9.5%)	**0.018**
	Agree	897 (85.7%)	1875 (77.9%)	**<0.001**	2206 (78.9%)	558 (86.8%)	**<0.001**	1340 (76.8%)	1432 (83.7%)	**<0.001**
**Symptomatic** **Infection**	Disagree	196 (18.7%)	467 (19.4%)	0.640	548 (19.6%)	106 (16.5%)	0.070	397 (22.8%)	266 (15.6%)	**<0.001**
	Not Sure	184 (17.6%)	609 (25.3%)	**<0.001**	675 (24.1%)	114 (17.7%)	**<0.001**	415 (23.8%)	378 (22.1%)	0.238
	Agree	667 (63.7%)	1331 (55.3%)	**<0.001**	1573 (56.3%)	423 (65.8%)	**<0.001**	932 (53.4%)	1066 (62.3%)	**<0.001**
**Community** **Transmission**	Disagree	178 (17.0%)	467 (19.4%)	0.096	521 (18.6%)	116 (18.0%)	0.727	400 (22.9%)	245 (14.3%)	**<0.001**
	Not Sure	169 (16.1%)	540 (22.4%)	**<0.001**	599 (21.4%)	108 (16.8%)	**0.009**	366 (21.0%)	343 (20.1%)	0.500
	Agree	700 (66.9%)	1400 (58.2%)	**<0.001**	1676 (59.9%)	419 (65.2%)	**0.014**	978 (56.1%)	1122 (65.6%)	**<0.001**
**Mutations Control**	Disagree	217 (20.7%)	372 (15.5%)	**<0.001**	443 (15.8%)	144 (22.4%)	**<0.001**	329 (18.9%)	260 (15.2%)	**0.004**
	Not Sure	152 (14.5%)	466 (19.4%)	**<0.001**	515 (18.4%)	100 (15.6%)	0.087	320 (18.3%)	298 (17.4%)	0.480
	Agree	678 (64.8%)	1569 (65.2%)	0.808	1838 (65.7%)	399 (62.1%)	0.077	1095 (62.8%)	1152 (67.4%)	**0.005**
**Equal Safety**	Disagree	68 (6.5%)	173 (7.2%)	0.463	203 (7.3%)	33 (5.1%)	0.054	145 (8.3%)	96 (5.6%)	**0.002**
	Not Sure	94 (9.0%)	476 (19.8%)	**<0.001**	508 (18.2%)	58 (9.0%)	**<0.001**	311 (17.8%)	259 (15.1%)	**0.033**
	Agree	885 (84.5%)	1758 (73.0%)	**<0.001**	2085 (74.6%)	552 (85.8%)	**<0.001**	1288 (73.9%)	1355 (79.2%)	**<0.001**
**Non-inferior Safety**	Disagree	689 (65.8%)	1240 (51.5%)	**<0.001**	1467 (52.5%)	453 (70.5%)	**<0.001**	956 (54.8%)	973 (56.9%)	0.217
	Not Sure	242 (23.1%)	852 (35.4%)	**<0.001**	955 (34.2%)	134 (20.8%)	**<0.001**	555 (31.8%)	539 (31.5%)	0.848
	Agree	116 (11.1%)	315 (13.1%)	0.101	374 (13.4%)	56 (8.7%)	**0.001**	233 (13.4%)	198 (11.6%)	0.113
**Risk-benefit Ratio**	Disagree	86 (8.2%)	245 (10.2%)	0.071	272 (9.7%)	52 (8.1%)	0.199	198 (11.4%)	133 (7.8%)	**<0.001**
	Not Sure	80 (7.6%)	346 (14.4%)	**<0.001**	383 (13.7%)	42 (6.5%)	**<0.001**	248 (14.2%)	178 (10.4%)	**<0.001**
	Agree	881 (84.1%)	1816 (75.4%)	**<0.001**	2141 (76.6%)	549 (85.4%)	**<0.001**	1298 (74.4%)	1399 (81.8%)	**<0.001**
**Self-prioritization**	Disagree	142 (13.6%)	483 (20.1%)	**<0.001**	537 (19.2%)	83 (12.9%)	**<0.001**	387 (22.2%)	238 (13.9%)	**<0.001**
	Not Sure	145 (13.8%)	364 (15.1%)	0.332	414 (14.8%)	92 (14.3%)	0.747	291 (16.7%)	218 (12.7%)	**0.001**
	Agree	760 (72.6%)	1560 (64.8%)	**<0.001**	1845 (66.0%)	468 (72.8%)	**<0.001**	1066 (61.1%)	1254 (73.3%)	**<0.001**
**Global Vaccine** **Justice**	Disagree	244 (23.3%)	808 (33.6%)	**<0.001**	896 (32.0%)	149 (23.2%)	**<0.001**	629 (36.1%)	423 (24.7%)	**<0.001**
	Not Sure	362 (34.6%)	893 (37.1%)	0.156	1058 (37.8%)	193 (30.0%)	**<0.001**	596 (34.2%)	659 (38.5%)	**0.008**
	Agree	441 (42.1%)	706 (29.3%)	**<0.001**	842 (30.1%)	301 (46.8%)	**<0.001**	519 (29.8%)	628 (36.7%)	**<0.001**
**National Vaccine Justice**	Disagree	330 (31.5%)	967 (40.2%)	**<0.001**	1099 (39.3%)	191 (29.7%)	**<0.001**	766 (43.9%)	531 (31.1%)	**<0.001**
	Not Sure	323 (30.9%)	830 (34.5%)	**0.037**	961 (34.4%)	188 (29.2%)	**0.013**	513 (29.4%)	640 (37.4%)	**<0.001**
	Agree	394 (37.6%)	610 (25.3%)	**<0.001**	736 (26.3%)	264 (41.1%)	**<0.001**	465 (26.7%)	539 (31.5%)	**0.002**
**Vaccine** **Satisfaction**	Disagree	393 (37.5%)	1012 (42.0%)	**0.013**	1151 (41.2%)	248 (38.6%)	0.227	764 (43.8%)	641 (37.5%)	**<0.001**
	Not Sure	531 (50.7%)	1149 (47.7%)	0.107	1370 (49.0%)	303 (47.1%)	0.391	786 (45.1%)	894 (52.3%)	**<0.001**
	Agree	123 (11.7%)	246 (10.2%)	0.182	275 (9.8%)	92 (14.3%)	**<0.001**	194 (11.1%)	175 (10.2%)	0.397
**Vaccine** **Selectivity**	Disagree	304 (29.0%)	767 (31.9%)	0.098	875 (31.3%)	190 (29.5%)	0.388	629 (36.1%)	442 (25.8%)	**<0.001**
	Not Sure	504 (48.1%)	1140 (47.4%)	0.675	1346 (48.1%)	292 (45.4%)	0.212	790 (45.3%)	854 (49.9%)	**0.006**
	Agree	239 (22.8%)	500 (20.8%)	0.176	575 (20.6%)	161 (25.0%)	**0.013**	325 (18.6%)	414 (24.2%)	**<0.001**
**Preferred** **Vaccine**	BTN162b2	189 (66.3%)	422 (70.7%)	0.188	476 (70.0%)	132 (66.7%)	0.371	257 (63.9%)	354 (73.8%)	**0.002**
	mRNA-1273	68 (23.9%)	125 (20.9%)	0.326	147 (21.6%)	45 (22.7%)	0.740	105 (26.1%)	88 (18.3%)	**0.005**
	AZD1222	11 (3.9%)	10 (1.7%)	**0.047**	11 (1.6%)	10 (5.1%)	0.013 *	9 (2.2%)	12 (2.5%)	0.800
	Ad26.COV2.S	17 (6.0%)	40 (6.7%)	0.678	46 (6.8%)	11 (5.6%)	0.543	31 (7.7%)	26 (5.4%)	0.167

Chi-squared test (*χ*^2^) and Fisher’s exact test (*) had been used with a significance level (*Sig*.) ≤ 0.05. The significant associations are in **bold** font.

**Table 7:** Determinants of COVID-19 Vaccine-related Attitudes among Czech Healthcare Workers Responding to COVID-19 Vaccine BD Survey, November 2021 (*n* = 3454).

	Variable	Outcome	Rejection (*n* = 570; 16.5%)	*Sig.*	Hesitancy (*n* = 420; 12.2%)	*Sig.*	Acceptance (*n* = 2464; 71.3%)	*Sig.*
Demographic	Gender	Female †	468 (16.7%)	0.159	379 (13.6%)	**<0.001**	1949 (69.7%)	**<0.001**
		Male	93 (14.5%)		40 (6.2%)		510 (79.3%)	
	† Pregnancy	Yes	12 (48.0%)	**<0.001**	4 (16.0%)	0.720	9 (36.0%)	**<0.001**
		No	456 (16.5%)		375 (13.5%)		1940 (70.0%)	
	Age Group	≤47 years-old	344 (19.7%)	**<0.001**	244 (14.0%)	**<0.001**	1156 (66.3%)	**<0.001**
		>47 years-old	226 (13.2%)		176 (10.3%)		1308 (76.5%)	
	Profession	Medical Professionals	154 (14.7%)	0.061	80 (7.6%)	**<0.001**	813 (77.7%)	**<0.001**
		Allied Health Professionals	416 (17.3%)		340 (14.1%)		1651 (68.6%)	
COVID-19-related Anamnesis	Infection	Yes ‡	268 (24.2%)	**<0.001**	180 (16.3%)	**<0.001**	658 (59.5%)	**<0.001**
		No	302 (12.9%)		240 (10.2%)		1806 (76.9%)	
	‡ Onset	Before 1st Dose	247 (25.4%)	**0.012**	155 (16.0%)	0.451	569 (58.6%)	0.104
		Between Doses	9 (13.0%)	**0.025**	16 (23.2%)	0.108	44 (63.8%)	0.455
		After 2nd Dose	12 (18.2%)	0.237	9 (13.6%)	0.549	45 (68.2%)	0.138
	‡ Severity	Asymptomatic	19 (24.1%)	0.969	9 (11.4%)	0.222	51 (64.6%)	0.341
		Mild	163 (24.8%)	0.611	106 (16.1%)	0.857	389 (59.1%)	0.758
		Moderate	81 (23.3%)	0.641	63 (18.2%)	0.252	203 (58.5%)	0.649
		Severe	3 (16.7%)	0.586 *	2 (11.1%)	0.753 *	13 (72.2%)	0.267
		Critical	2 (50.0%)	0.249 *	0 (0%)	1.000 *	2 (50.0%)	1.000 *
	‡ Signs & Symptoms	Fever	153 (24.5%)	0.799	94 (15.1%)	0.215	377 (60.4%)	0.477
		Cough	133 (24.1%)	0.915	96 (17.4%)	0.315	323 (58.5%)	0.508
		Dyspnea	73 (22.8%)	0.482	53 (16.6%)	0.869	194 (60.6%)	0.625
		Fatigue	210 (24.6%)	0.581	155 (18.2%)	**0.002**	488 (57.2%)	**0.004**
		Myalgia	175 (23.9%)	0.725	127 (17.3%)	0.175	430 (58.7%)	0.477
		Headache	176 (25.1%)	0.353	128 (18.3%)	**0.017**	396 (56.6%)	**0.009**
		Anosmia	170 (25.6%)	0.181	120 (18.1%)	**0.044**	373 (56.3%)	**0.007**
		Dysgeusia	129 (25.2%)	0.466	97 (19.0%)	**0.024**	285 (55.8%)	**0.019**
		Pharyngitis	56 (27.7%)	0.200	33 (16.3%)	0.979	113 (55.9%)	0.255
		Congestion	99 (27.1%)	0.115	73 (20.0%)	**0.019**	193 (52.9%)	**0.002**
		Rhinitis	102 (28.7%)	**0.018**	67 (18.8%)	0.114	187 (52.5%)	**0.001**
		Nausea	36 (22.9%)	0.681	26 (16.6%)	0.917	95 (60.5%)	0.780
		Vomiting	10 (18.2%)	0.283	13 (23.6%)	0.129	32 (58.2%)	0.839
		Diarrhea	43 (21.0%)	0.228	41 (20.0%)	0.109	121 (59.0%)	0.879
Vaccine-related Anamnesis	Vaccinated	Yes Ψ	422 (12.8%)	**<0.001**	408 (12.4%)	**0.044**	2457 (74.7%)	**<0.001**
		No	148 (88.6%)		12 (7.2%)		7 (4.2%)	
	Ψ Vaccine Type	BTN162b2	349 (11.7%)	**<0.001**	349 (11.7%)	**<0.001**	2282 (76.6%)	**<0.001**
		mRNA-1273	35 (20.0%)	**0.004**	44 (25.1%)	**<0.001**	96 (54.9%)	**<0.001**
		AZD1222	11 (12.5%)	0.923	10 (11.4%)	0.762	67 (76.1%)	0.761
		Ad26.COV2.S	27 (61.4%)	**<0.001**	5 (11.4%)	0.823	12 (27.3%)	**<0.001**
	Ψ Doses Number	One	29 (49.2%)	**<0.001**	13 (22.0%)	0.024	17 (28.8%)	**<0.001**
		Two	291 (17.8%)	**<0.001**	366 (22.4%)	**<0.001**	976 (59.8%)	**<0.001**
		Three	102 (6.4%)	**<0.001**	29 (1.8%)	**<0.001**	1464 (91.8%)	**<0.001**
	Ψ Medical Care	Yes	48 (42.5%)	**<0.001**	22 (19.5%)	**0.021**	43 (38.1%)	**<0.001**
		No	374 (11.8%)		386 (12.2%)		2414 (76.1%)	
Psychosocial Drivers of COVID-19 Vaccine BD-related Attitudes	Severe Illness	Disagree	246 (79.6%)	**<0.001**	29 (9.4%)	0.118	34 (11.0%)	**<0.001**
		Not Sure	111 (29.8%)	**<0.001**	193 (51.7%)	**<0.001**	69 (18.5%)	**<0.001**
		Agree	213 (7.7%)	**<0.001**	198 (7.1%)	**<0.001**	2361 (85.2%)	**<0.001**
	Symptomatic Infection	Disagree	302 (45.6%)	**<0.001**	101 (15.2%)	**0.007**	260 (39.2%)	**<0.001**
		Not Sure	129 (16.3%)	0.839	213 (26.9%)	**<0.001**	451 (56.9%)	**<0.001**
		Agree	139 (7.0%)	**<0.001**	106 (5.3%)	**<0.001**	1753 (87.7%)	**<0.001**
	Community Transmission	Disagree	330 (51.2%)	**<0.001**	124 (19.2%)	**<0.001**	191 (29.6%)	**<0.001**
		Not Sure	102 (14.4%)	0.089	190 (26.8%)	**<0.001**	417 (58.8%)	**<0.001**
		Agree	138 (6.6%)	**<0.001**	106 (5.0%)	**<0.001**	1856 (88.4%)	**<0.001**
	Mutations Control	Disagree	111 (18.8%)	0.093	37 (6.3%)	**<0.001**	441 (74.9%)	**0.037**
		Not Sure	77 (12.5%)	**0.003**	87 (14.1%)	0.107	454 (73.5%)	0.197
		Agree	382 (17.0%)	0.282	296 (13.2%)	**0.013**	1569 (69.8%)	**0.007**
	Equal Safety	Disagree	179 (74.3%)	**<0.001**	25 (10.4%)	0.379	37 (15.4%)	**<0.001**
		Not Sure	166 (29.1%)	**<0.001**	175 (30.7%)	**<0.001**	229 (40.2%)	**<0.001**
		Agree	225 (8.5%)	**<0.001**	220 (8.3%)	**<0.001**	2198 (83.2%)	**<0.001**
	Non-inferior Safety	Disagree	184 (9.5%)	**<0.001**	118 (6.1%)	**<0.001**	1627 (84.3%)	**<0.001**
		Not Sure	232 (21.2%)	**<0.001**	233 (21.3%)	**<0.001**	629 (57.5%)	**<0.001**
		Agree	154 (35.7%)	**<0.001**	69 (16.0%)	**0.009**	208 (48.3%)	**<0.001**
	Risk-benefit Ratio	Disagree	251 (75.8%)	**<0.001**	27 (8.2%)	**0.019**	53 (16.0%)	**<0.001**
		Not Sure	130 (30.5%)	**<0.001**	206 (48.4%)	**<0.001**	90 (21.1%)	**<0.001**
		Agree	189 (7.0%)	**<0.001**	187 (6.9%)	**<0.001**	2321 (86.1%)	**<0.001**
	Self-prioritization	Disagree	298 (47.7%)	**<0.001**	119 (19.0%)	**<0.001**	208 (33.3%)	**<0.001**
		Not Sure	99 (19.4%)	**0.052**	146 (28.7%)	**<0.001**	264 (51.9%)	**<0.001**
		Agree	173 (7.5%)	**<0.001**	155 (6.7%)	**<0.001**	1992 (85.9%)	**<0.001**
	Global Vaccine Justice	Disagree	360 (34.2%)	**<0.001**	209 (19.9%)	**<0.001**	483 (45.9%)	**<0.001**
		Not Sure	123 (9.8%)	**<0.001**	157 (12.5%)	0.634	975 (77.7%)	**<0.001**
		Agree	87 (7.6%)	**<0.001**	54 (4.7%)	**<0.001**	1006 (87.7%)	**<0.001**
	National Vaccine Justice	Disagree	374 (28.8%)	**<0.001**	232 (17.9%)	**<0.001**	691 (53.3%)	**<0.001**
		Not Sure	124 (10.8%)	**<0.001**	136 (11.8%)	0.643	893 (77.5%)	**<0.001**
		Agree	72 (7.2%)	**<0.001**	52 (5.2%)	**<0.001**	880 (87.6%)	**<0.001**
	Vaccine Satisfaction	Disagree	227 (16.2%)	0.650	158 (11.2%)	0.173	1020 (72.6%)	0.175
		Not Sure	278 (16.5%)	0.945	212 (12.6%)	0.422	1190 (70.8%)	0.524
		Agree	65 (17.6%)	0.542	50 (13.6%)	0.387	254 (68.8%)	0.261
	Vaccine Selectivity	Disagree	220 (20.5%)	**<0.001**	130 (12.1%)	0.979	721 (67.3%)	**<0.001**
		Not Sure	255 (15.5%)	0.135	203 (12.3%)	0.747	1186 (72.1%)	0.320
		Agree	95 (12.9%)	**0.003**	87 (11.8%)	0.716	557 (75.4%)	**0.006**
	Preferred Vaccine	BTN162b2	69 (11.3%)	**<0.001**	65 (10.6%)	0.255	477 (78.1%)	**<0.001**
		mRNA-1273	22 (11.4%)	0.248	24 (12.4%)	0.627	147 (76.2%)	0.573
		AZD1222	5 (23.8%)	0.187	2 (9.5%)	0.779	14 (66.7%)	0.398
		Ad26.COV2.S	27 (47.4%)	**<0.001**	10 (17.5%)	0.135	20 (35.1%)	**<0.001**

Chi-squared test (*χ*^2^) and Fisher’s exact test (*) had been used with a significance level (*Sig*.) ≤ 0.05. † Female participants. ‡ Pregnant participants. The significant associations are in **bold** font.

**Table 8:** Regression Analysis of COVID-19 Vaccine Hesitancy *vs.* Acceptance Demographic and Anamnestic Determinants among Czech Healthcare Workers Responding to COVID-19 Vaccine BD Survey, November 2021 (*n* = 3454).

Hesitancy					Acceptance				
Predictor	B (SE)	Wald	OR (CI 95%)	*Sig.*	Predictor	B (SE)	Wald	OR (CI 95%)	*Sig.*
Female (*vs.* Male)	0.86 (0.17)	24.91	2.36 (1.69–3.31)	**<0.001**	Male (*vs.* Female)	0.51 (0.11)	23.34	1.67 (1.36–2.05)	**<0.001**
Pregnancy: Yes (*vs.* No)	0.20 (0.55)	0.13	1.22 (0.42–3.57)	0.720	Pregnancy: No (*vs.* Yes)	1.42 (0.42)	11.55	4.15 (1.83–9.43)	**<0.001**
≤47 yo (*vs*. >47 yo)	0.35 (0.11)	10.98	1.42 (1.15–1.74)	**<0.001**	>47 yo (*vs*. ≤47 yo)	0.50 (0.08)	43.63	1.66 (1.43–1.92)	**<0.001**
AHP (*vs.* Medical)	0.69 (0.13)	27.85	1.99 (1.54–2.57)	**<0.001**	Medical (*vs.* AHP)	0.46 (0.09)	29.01	1.59 (1.34–1.88)	**<0.001**
Infection: Yes (*vs.* No)	0.54 (0.11)	25.38	1.71 (1.39–2.10)	**<0.001**	Infection: No (*vs.* Yes)	0.82 (0.08)	109.11	2.27 (1.95–2.65)	**<0.001**
Vaccinated: No (*vs.* Yes)	0.61 (0.30)	3.95	1.83 (1.01–3.32)	**0.047**	Vaccinated: Yes (*vs.* No)	4.22 (0.39)	228.85	67.66 (31.62–144.81)	**<0.001**
Care: Yes (*vs.* No)	0.56 (0.24)	5.23	1.75 (1.08–2.82)	**0.022**	Care: No (*vs.* Yes)	1.64 (0.20)	68.74	5.17 (3.51–7.63)	**<0.001**

Binary logistic regression had been used with a significance level (*Sig*.) ≤ 0.05. AHP refers to Allied Health Professionals. The significant associations are in **bold** font.

**Table 9:** Regression Analysis of COVID-19 Vaccine Acceptance Psychosocial Determinants among Czech Healthcare Workers Responding to COVID-19 Vaccine BD Survey, November 2021 (*n* = 3454).

Predictor	B (SE)	Wald	AOR (CI 95%)	*Sig.*
**Severe Illness**: Agree	3.24 (0.14)	542.23	25.55 (19.45–33.57)	**<0.001**
**Symptomatic Infection**: Agree	1.76 (0.10)	310.51	5.81 (4.78–7.07)	**<0.001**
**Community Transmission**: Agree	2.07 (0.10)	410.49	7.90 (6.47–9.65)	**<0.001**
**Mutations Control**: Disagree	0.27 (0.13)	4.21	1.31 (1.01–1.69)	**0.040**
**Equal Safety**: Agree	1.99 (0.11)	350.53	7.32 (5.94–9.01)	**<0.001**
**Non-inferior Safety**: Disagree	1.38 (0.10)	198.93	3.97 (3.28–4.81)	**<0.001**
**Risk-benefit Ratio**: Agree	2.97 (0.13)	565.22	19.42 (15.20–24.80)	**<0.001**
**Self-prioritization**: Agree	1.96 (0.10)	383.86	7.10 (5.83–8.63)	**<0.001**
**Global Vaccine Justice**: Disagree	−1.46 (0.10)	226.91	0.23 (0.19–0.28)	**<0.001**
**National Vaccine Justice**: Disagree	−1.14 (0.09)	146.96	0.32 (0.27–0.39)	**<0.001**
**Vaccine Satisfaction**: Agree	0.06 (0.16)	0.16	1.06 (0.78–1.44)	0.689
**Vaccine Selectivity**: Agree	0.14 (0.11)	1.49	1.15 (0.92–1.44)	0.223

Binary logistic regression had been used with a significance level (*Sig*.) ≤ 0.05 and adjusted for gender, pregnancy, age, profession, COVID-19 infection and vaccination, and seeking medical care. The significant associations are in **bold** font.

## Data Availability

The data that support the findings of this study are available from the corresponding author upon reasonable request.

## Supplementary Materials

The following are available online at https://www.mdpi.com/article/10.3390/vaccines9121437/s1, Figure S1. The sample size of healthcare workers in Czech Republic—Epi-InfoTM version 7.2.4. Table S1. The results of test re-test reliability. Table S2. Demographic Characteristics of Czech Healthcare Workers Responding to COVID-19 Vaccine BD Survey, November 2021 (*n* = 3454).
