# Supplementary material for: COVID-19 Vaccine Booster Hesitancy (VBH) of Healthcare Workers in Czechia: National Cross-Sectional Study
Source: Vaccines (Basel). 2021 Dec 6;9(12):1437. doi: 10.3390/vaccines9121437 (PMC8705445; doi:10.3390/vaccines9121437)
Supplement: Supplementary file 1 [file vaccines-09-01437-s001.zip › vaccines-1491656-supplementary.pdf]

vaccines

*Supplementary File*

# COVID-19 Vaccine Booster Hesitancy (VBH) of Healthcare Workers in Czechia: National Cross-sectional Study

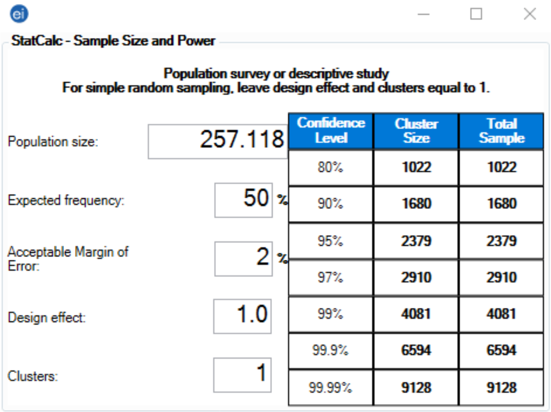


**Figure S1. The sample size of healthcare workers in Czech Republic—Epi-Info™ version 7.2.4.**

**Table S1. The results of test re-test reliability.**

| Participant | κ coefficient | Participant | κ coefficient |
|---|---|---|---|
| No. 1 | 0.77 | No. 5 | 1.00 |
| No. 2 | 0.85 | No. 6 | 1.00 |
| No. 3 | 0.69 | No. 7 | 0.55 |
| No. 4 | 0.57 | No. 8 | 1.00 |

Cohen's Kappa statistic ($\kappa$); 0.01 – 0.20 as none to slight, 0.21 – 0.40 as fair, 0.41 – 0.60 as moderate, 0.61–0.80 as substantial, and 0.81–1.00 as perfect agreement [1].

**Table S2. Demographic Characteristics of Czech Healthcare Workers Responding to COVID-19 Vaccine BD Survey, November 2021 ($n$ = 3454).**

| Variable | Outcome | Frequency ($n$) | Percentage (%) |
|---|---|---|---|
| **Gender** | Female † | 2796 | 80.9 % |
| | Male | 643 | 18.6 % |
| | LGBTQ+ | 15 | 0.4 % |
| **† Pregnancy** | Yes ‡ | 25 | 0.9 % |
| | No | 2771 | 99.1 % |
| **‡ Trimester** | 1st Trimester | 9 | 36 % |
| | 2nd Trimester | 10 | 40 % |
| | 3rd Trimester | 6 | 24 % |
| **Age** | ≤ 47 years-old | 1744 | 50.5 % |
| | > 47 years-old | 1710 | 49.5 % |
| **Profession (Medical)** | Physician | 980 | 28.4 % |
| | Dentist | 14 | 0.4 % |
| | Pharmacist | 53 | 1.5 % |
| **Profession (Allied Health)** | General Nurse | 1452 | 42 % |
| | Paediatric Nurse | 150 | 4.3 % |
| | Midwife | 90 | 2.6 % |
| | Paramedic | 28 | 0.8 % |

| | | | |
|---|---|---|---|
| | Physiotherapist | 85 | 2.5 % |
| | Nutrition Therapist | 57 | 1.7 % |
| | Occupational Therapist | 10 | 0.3 % |
| | Speech Therapist | 7 | 0.2 % |
| | Psychologist | 30 | 0.9 % |
| | Addictologist | 8 | 0.2 % |
| | Pharmaceutical Assistant | 103 | 3.0 % |
| | Medical Laboratory Technician | 145 | 4.2 % |
| | Radiology Assistant | 69 | 2.0 % |
| | Radiology Physicist | 11 | 0.3 % |
| | Biomedical Engineer | 24 | 0.7 % |
| | Biomedical Technician | 3 | 0.1 % |
| | Specialist in Public Health | 15 | 0.4 % |
| | Specialist in Laboratory Methods | 74 | 2.1 % |
| | Specialist in Healthcare Management | 36 | 1.0 % |
| | Public Health Assistant | 6 | 0.2 % |
| | Dental Technician | 4 | 0.1 % |
| **Region** | Central Bohemian | 337 | 9.8 % |
| | Prague | 1010 | 29.2 % |
| | South Moravian | 699 | 20.2 % |
| | Moravian-Silesian | 208 | 6.0 % |
| | Usti nad Labem | 185 | 5.4 % |
| | South Bohemian | 74 | 2.1 % |
| | Olomouc | 124 | 3.6 % |
| | Plzen | 90 | 2.6 % |
| | Zlín | 117 | 3.4 % |
| | Hradec Kralove | 152 | 1.7 % |
| | Pardubice | 154 | 4.5 % |
| | Vysočina | 139 | 4.0 % |
| | Liberec | 107 | 3.1 % |
| | Karlovy Vary | 58 | 4.4 % |

No missing data. † Female participants. ‡ Pregnant participants.

## References

1. McHugh, M.L. Interrater reliability: The kappa statistic. *Biochem. Medica* **2012**, *22*, 276–282, doi:10.11613/bm.2012.031.
